# Using routinely collected data to develop and evaluate a clinical tool for early identification of palliative care needs in long-term care: The RESPECT Project.

**DOI:** 10.23889/ijpds.v7i3.1858

**Published:** 2022-08-25

**Authors:** Amy T. Hsu, Cathryn Espadero, Peter Tanuseputro, Carol Bennett, Sarah Beach, Rhiannon Roberts, Douglas Manuel

**Affiliations:** 1 Bruyère Research Institute; 2 University of Ottawa; 3 Ottawa Hospital Research Institute

## Objectives

Prognostication tools reporting personalized mortality risk and survival can improve advance care planning and discussions about end-of-life care. We developed, validated, and implemented a mortality risk algorithm for older adults with diverse care needs in long-term care (LTC) homes, called the Risk Evaluation for Support: Predictions for Elder-Life in the Community Tool for LTC (RESPECT-LTC).

## Approach

RESPECT-LTC was developed using routinely-collected health information on residents in LTC homes in Ontario, Canada. Model development used a cohort of LTC residents aged 50 years or older with at least 1 Resident Assessment Instrument—Minimum Data Set (RAI-MDS) record between January 2010 and December 2016. The primary outcome was mortality 6 months after a RAI-MDS assessment. We used proportional hazards regression with robust standard errors to account for clustering by the individual. We validated this algorithm, temporally, in a cohort of LTC residents who were assessed between January and December 2017. We constructed 37 risk bins based on incremental increases in estimated median survival of ~3 weeks among residents at high risk of death and 3 months among residents with lower mortality risk. We implemented and are evaluating the use of RESPECT-LTC for early identification of palliative care needs in LTC homes across Ontario.

## Results

Development and validation cohorts included 2,228,176 and 328,204 RAI-MDS assessments, respectively. Mean predicted 6-month mortality risk ranged from 1.38% (95% CI 0.63%-1.61%) in the lowest to 91.97% (95% CI 81.47%-99.9%) in the highest risk group. Estimated median survival spanned from 42 days (15 to 128 d at the 25th and 75th percentiles) in the highest risk group to over 8 years (2,066 to 3,428 d) in the lowest risk group. The algorithm had a c-statistic of 0.730 (95% CI 0.726–0.736) in our validation cohort.

## Conclusion and Relevance

RESPECT-LTC makes use of routinely-collected information to improve the identification of palliative and end-of-life care needs in LTC. Ongoing evaluation will assess its impact on referrals to palliative care, hospitalization at the end of life, and location of death.

